# Chronic Prurigo: Similar Clinical Profile and Burden Across Clinical Phenotypes

**DOI:** 10.3389/fmed.2021.649332

**Published:** 2021-06-29

**Authors:** Claudia Zeidler, Manuel Pedro Pereira, Sonja Ständer

**Affiliations:** Department of Dermatology and Center for Chronic Pruritus, University Hospital Münster, Münster, Germany

**Keywords:** prurigo nodularis, pruritus, burden, quality of life, itch characteristics

## Abstract

Chronic prurigo is a debilitating skin disease characterized by the presence of chronic pruritus and scratching-related pruriginous lesions. The pruriginous lesions can differ in their clinics what has recently been categorized into different clinical phenotypes. The most common one is chronic nodular prurigo (syn. prurigo nodularis); other phenotypes are papular, plaque, umbilicated, and linear prurigo. A comparison between these phenotypes regarding similarities and differences has not yet been performed. In this explorative analysis, itch characteristics, scratching behavior, and disease burden of the nodular, papular, plaque, and umbilicated prurigo were investigated in 1,128 patients. Patients with nodular and plaque prurigo were younger than patients with papular and umbilicated prurigo. The shortest duration of the underlying pruritus was found in papular and umbilicated prurigo, the longest in plaque prurigo. Itch intensity, impairment of sleep, mood and the quality of life did not differ. These findings confirm that the clinical phenotypes of chronic prurigo belong to a spectrum of one disease with similar disease characteristics and can be categorized under the umbrella term of chronic prurigo. Future clinical trials should include all phenotypes of chronic prurigo.

## Introduction

**Figure 1:**
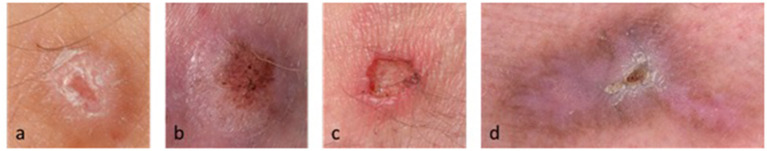
Different types of pruriginous lesions. **(a)** papules (<1cm), **(b)** nodules (≥1cm), **(c)** ulcerated pruriginous lesions, **(d)** plaques.

Chronic prurigo (CPG) is a rare but worldwide occurring disease (1, 2). It is characterized by the presence of chronic pruritus (CP), history, and/or evidence of repeated scratching (e.g., excoriations, scars) as well as localized or generalized pruriginous lesions (3). The clinical picture differs in the number, distribution and the type of the pruriginous lesions such as papules, nodules, and/or plaques (3, 9, 10). However, multiple types of pruriginous lesions can be present in the same patient. These lesions dot not allow a conclusion to the underlying etiology of CPG. Depending on the predominant pruriginous lesion in one patient (Figure 1), the CPG is divided into the following clinical phenotypes according to the disease definition (3): chronic nodular prurigo (nodules predominate), chronic papular prurigo (papules predominate), chronic plaque prurigo (plaques predominate), and chronic umbilicated prurigo (ulcerated pruriginous lesions predominate) (3). As these phenotypes fulfill the definition of CPG, it was proposed that CPG is an umbrella term for the various clinical presentations (3).

In the past, studies in CPG focused on analyzing itch characteristics and disease burden exclusively in chronic nodular prurigo. From these studies it is known that patients with chronic nodular prurigo have a high burden and a substantial impairment of the quality of life, even when compared to patients with CP on non-lesional skin (4, 5). Sleep quality can be severely limited in these patients and the presence of psychological comorbidities can frequently occur in patients with chronic nodular prurigo (5, 6). However, until now, no study included the other clinical phenotypes of CPG (papular, plaque, and umbilicated type) and it is unknown if the aggregation of these phenotypes under one disease is justified and if there are biological differences between the clinical phenotypes of CPG.

Therefore, this study aims detecting possible differences between the CPG phenotypes regarding clinical characteristics using a retrospective approach and to verify the suggestion that the clinical phenotypes can categorized under the umbrella term of CPG.

## Materials and Methods

### Data Source

Data from routine care were used to perform this retrospective analysis. All data from patients of the Center for Chronic Pruritus were transferred into a biomedical data base. After written informed consent, data were collected by the physicians (i.e., medical history and physical examination), and from several patient reported outcome tools (e.g., pruritus questionnaire, quality of life). Afterwards the data were reviewed by the physician-in-chief and transferred to the research database of the Center for Chronic Pruritus at the University Hospital Münster, which is approved by the Ethics Committee of the Medical Faculty of the University of Münster (2007-413-f-S) (7). For this analysis, data of the first consultation of patients with CPG, who presented between October 2004 and February 2018, were included.

### Etiology of CPG

According to the International Forum for the Study of Itch (IFSI) (8) the etiology of CPG was diagnosed and classified by a physician.

### Patient Reported Outcome Tools

The numerical rating scale (NRS), ranging from 0 (no pruritus) to 10 (worst imaginable pruritus) was used to assess itch intensity of the previous 24 h (worst itch intensity in the last 24 h = WI-NRS/24 h; average itch intensity of the last 24 h = AI-NRS/24 h). The “Neuroderm” questionnaire, a pruritus questionnaire, was used to assess the scratching behavior and the impact on sleep (9). The impairment of the quality of life was assessed by using the “Dermatology Life Quality Index (DLQI)” (10) and the possible presence of an anxiety and/or a depression was investigated using the screening questionnaire “Hospital Anxiety and Depression Scale (HADS)” (11).

### Statistical Analyses

For each metric endpoint, the Hodges-Lehmann estimate of the median with two-sided 95% confidence interval (CI) was calculated by using the R function wilcox.exact from the R package exactRankTests. For each binary endpoint, the absolute numbers and percentages per category were calculated for each group. For a chosen reference, an exact Clopper-Pearson 95% CI for the percentage was calculated by using the R function exactci from the R package PropCIs. All analyses are of explorative character. No adjustments for multiple testing were performed. If the CIs of the groups did not differ considerably, it was interpreted as an indication of equivalence. All statistical analyses were predetermined in a statistical analysis plan and were performed by R 3.4.4.

## Results

1,128 patients with CPG [692 (61.4%) female] were included in this study. Most of them suffered from chronic nodular prurigo [*n* = 908 (80.5%)] (Table 1). Patients with nodular prurigo and plaque prurigo were younger than patients with papular or umbilicated prurigo (Table 1). Nodular, popular, and plaque prurigo predominantly affected women (60.6-66.7%); in the group of patients suffering from umbilicated prurigo, men were more affected (66.7%).

**Table 1:** Demographics, duration and origin of chronic pruritus and burden of disease in the different clinical phenotypes.

	**Nodular** **Prurigo**	**Popular** **prurigo**	**Umbilicated** **prurigo**	**Plaque** **prurigo**	**Differences**
**Number**					
*N*	908	193	24	3	Plaque < umbilicated < papular < nodular prurigo
%	80.5	17.1	2.1	0.3	
**Age (yrs)**					
Median	64.3	71	73	60.3	Plaque < nodular < papular < umbilicated prurigo
95% CI	62.8-65.8	66-73.4	66.8-77.8	52.2-64.7	
**Sex**					
Male, *n* (%)	343 (37.8)	76 (39.4)	16 (66.7)	1 (33.3)	More females affected except in umbilicated prurigo.
Female, *n* (%)	565 (62.2)	117 (60.6)	8 (33.3)	2 (66.7)	
**Duration of pruritus (yrs)**					
Median	3.5	1.86	1.64	6	Umbilicated < papular < nodular < plaque prurigo
95% CI	3.03-4.11	1.59-2.05	1.08-3.04	6.4-10.2	
**Origin of pruritus (%)**					
Dermatologic	22.8	36.7	12.5	66.7	Most frequent origin per phenotype is highlighted in gray.
Systemic	9.0	8.8	20.8	0	
Neurologic	6.5	12.8	12.5	0	
Psychiatric	1.2	2.4	4.2	0	
Multifactorial	54.4	36.2	50	33.3	
Unknown	6.1	3.1	0	0	
**Intensity of pruritus**					
WI-NRS/24h					
Median	8.5	8.5	8.75	8	No differences according to 95% CI.
95% CI	8.5-9.0	8-9.0	7.5-9.0	6.5-8.0	
AI-NRS/24h					
Median	6.5	6.5	7.75	6.5	
95% CI	6.0-7.0	6.0-7.0	5.0-8.5	5.2-6.8	
**Scratching behavior (%)**					
Scratching only when itching	71.0	81.3	82.4	66.6	Positive outliers are highlighted in dark gray, negative outliers in light gray.
Scratching causes itch relief	68.8	47.0	47.1	33.3	
Scratching injures the skin	63.0	51.5	64.7	33.3	
Scratching is done unconsciously	51.2	29.5	29.4	33.3	
Scratching causes itch aggravation	49.3	36.6	23.5	33.3	
Scratching provides satisfaction	30.5	27.1	17.6	33.3	
Rubbing	64.9	67.9	41.1	66.6	
Chafing	25.6	20.1	5.9	33.3	
Pinching	16.5	10.5	17.6	33.3	
Kneading	7.8	9.0	5.9	33.3	
**Quality of life**					
DLQI					
Median	13.0	10.0	12.0	8.0	No differences according to 95% CI.
95% CI	11.0-15.5	9.2-13.5	9-12.7	7.4-12.0	
**Anxiety and depressions**					
HADS-A					
Median	7.0	7.0	8.0	7.0	No differences according to 95% CI.
95% CI	6.4-9.3	5.9-8.7	6.6-9.0	5.8-8.4	
HADS-D					
Median	7.0	6.0	8.5	5.0	
95% CI	7.0-9.2	6.0-8.1	8.5-9.4	5.0-7.4	
**Loss of slept hours/day**					
Median	3	3	3.5	2	No differences.
Mean	3.2	3	3	2.6	
SD(±)	2	1.8	1.8		

*Yrs, years; WI-NRS/24h, worst pruritus intensity in the last 24 h; AI-NRS/24 h, average pruritus intensity in the last 24 h; DLQI, Dermatology Life Quality Index; HADS-A, Hospital Anxiety and Depressions Scale for Anxiety; HADS-D, Hospital Anxiety and Depressions Scale for Depressions; SD, standard deviation*.

### Duration and Cause of Chronic Prurigo

Overall, the patients suffered from CP for a median duration of 2.9 years (CI 2.6-3.1) before presenting in our center. A comparison of the groups showed that patients with papular prurigo and umbilicated prurigo had the shortest, patients with nodular prurigo an intermediate and patients with plaque prurigo the longest duration from CP (Table 1).

The etiology of pruritus classified according to the IFSI classification (8) was most frequently found to be of multifactorial origin in nodular and umbilicated prurigo. The patients with papular and plaque prurigo showed most common a dermatosis as the cause of CP (Table 1). Dermatological diseases were the second most frequently found origins in patients with nodular prurigo, while in umbilicated prurigo the second most frequent origin was a systemic disease.

### Itch Intensity

The median AI-NRS/24h was 6.5/10, whereas the median WI-NRS/24h was 8.5 for the whole patient population. No differences between the phenotypes were found when comparing the pruritus intensity (Table 1).

### Scratching Behavior

**Figure 2:**
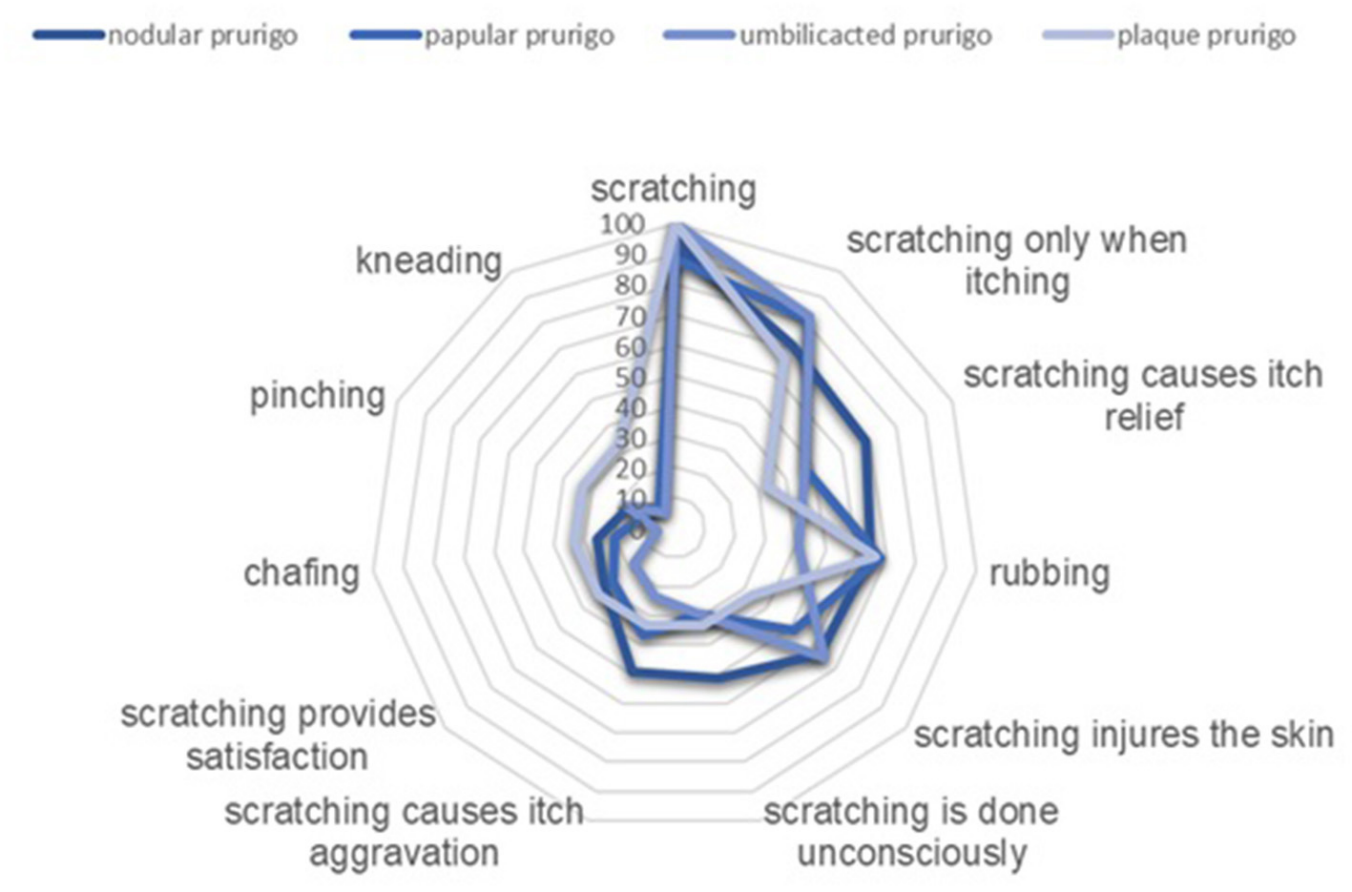
Comparison of scratching behavior in patients with different clinical phenotypes of chronic prurigo (in %).

Almost all patients (over 90%) reported that they scratch themselves, and that they were aware that scratching injures the skin (60.7%). Interestingly, 42.4% of all patients stated scratching can cause itch aggravation. Divided into the phenotypes, some differences could be found: Patients with nodular prurigo stated more frequently “scratching causes itch relief,” “scratching is done unconsciously,” and “scratching causes itch aggravation” (Figure 2).

There were no differences in the kind of scratching between patients with nodular and papular prurigo. Whereas in contrast to all other phenotypes patients with plaque prurigo reported more frequent to pinch and knead and patients with umbilicated prurigo do less rub and chafe their skin (Table 1).

### Burden of CPG

Patients reported sleep problems independent of the clinical phenotype. The loss of sleeping hours did not differ between the phenotypes. All patients achieved comparable values in the total score of the DLQI and the subscores for anxiety and depression in the HADS (Table 1).

## Discussion

The aim of this explorative study was to investigate the demographic profile, itch intensity, scratching behavior, and burden of the different clinical phenotypes of CPG. We found more similarities in the analyzed parameter than differences between phenotypes. For example, pruritus characteristics, the impact on sleep, quality of life and mood did not differ between the groups. Regarding pruritus intensity at baseline, the median of the average pruritus intensity in the last 24 h was classified as moderate to severe; the median of the worst pruritus intensity in the last 24 h as severe in all phenotypes. These results confirm analyzes of previous studies examining the pruritus intensity of patients with chronic nodular prurigo (4, 5). It can be speculated that as soon as the CPG has developed due to neuronal sensitization processes and the development of a vicious circle of experiencing pruritus and continuously scratching independent of the origin, the severity of the itch intensity in the different phenotypes is similar.

Differences were identified regarding the categorized etiology of CPG. A multifactorial etiolgy was prominent in nodular and umbilicated prurigo whereas more dermatological diseases were found in papular and plaque prurigo. Despite these differences in the categories, no conclusion is justified based on these data to make a differential diagnosis regarding the individual etiology of CPG in affected patients.

Another descriptive difference was found regarding the duration of the underlying pruritus in CPG. Patients with plaque prurigo suffered the longest, followed by patients with nodular prurigo and then papular and umbilicated type. It can thus be assumed that the duration of the CPG and the associated prolonged scratching contributes to different clinical phenotypes. This supports the assumption that some clinical phenotypes of CPG are a continuous spectrum of the disease and can merge. Also another earlier study suggested that the various pruriginous lesions such as papules, nodules and plaques represent stages of the development in CPG (12).

All patients have in common a severe scratching behavior with some differences which might be relevant for development of the different phenotypes. More than half of the patients with nodular prurigo scratch themselves unconsciously, while only a third of patients suffering from the other CPG phenotypes made this statement. Patients with umbilicated prurigo less often chafe or rub compared to all other phenotypes. However, there are also patients in these group who do chafing or rubbing their skin. This observation does not disambiguate that the way of scratching leads to a particular phenotype of CPG.

It was also noticeable that scratching was reported to cause both pruritus relief and aggravation. These statements, contradicting at first glance, reflect the vicious circle of itching and scratching that is a typical characteristic of CPG. Satisfaction and pruritus control by scratching may directly occur while or shortly after scratching, but the pruritus recurs again afterwards.

Comparing the burden of patients, the phenotypes of CPG showed no differences in the impairment of the quality of life, in the scores for screening for anxiety and depression and in restriction of sleep. The median values from the DLQI for nodular prurigo, umbilicated prurigo, and plaque prurigo were in the range that indicate a strong impairment of the quality of life, for papular prurigo with one point below the cut-off value in the range for moderate impairment. However, all confidence intervals are comparable, therefore no important differences can be assumed here. In the past, the use of DLQI in patients with nodular prurigo showed also a strong impairment of the quality of life (13–15). Compared to these analyses of the quality of life in patients with nodular prurigo, our findings in patients of different clinical phenotypes are identical.

## Conclusions

Overall, differences could be found in the age of the patients, in the duration and origin of the underlying CP and, though minimal, the scratching behavior. However, the itch intensity and the burden did not differ across the different clinical phenotypes of CPG. These findings confirm that clinical phenotypes of CPG are a spectrum and a part of the same disease, supporting the proposed term CPG as an umbrella term including all phenotypes of CPG. Based on these data and the assumed identical pathogenesis of the phenotypes, we suppose that the treatment response of patients with CPG is equal, regardless of the clinical phenotype. In the future, further studies should be carried out to confirm this statement and in order to find out whether new antipruritic treatments should be tested in each individual phenotype or in a cohort of CPG independent from its phenotype.

## Limitations

Since data were not normally distributed, we did not perform comparisons of distributions to calculate *p*-values in this exploratory analysis. We rather examined if the 95% CI's differed from each other for each variable. If this was not the case, it was interpreted as an indication of equivalence. The differences to plaque prurigo should be interpreted carefully, as very few patients suffered from it.

## Data Availability Statement

The datasets presented in this article are not readily available. Requests to access the datasets should be directed to Claudia Zeidler, claudia.zeidler@ukmuenster.de.

## Author Contributions

All authors listed have made a substantial, direct and intellectual contribution to the work, and approved it for publication.

## Conflict of Interest

CZ has received speaker honoraria/travel fees from Beiersdorf and Dermasence. MP is an investigator for Trevi Therapeutics; is a consultant for Galderma; and has received speaker honoraria/travel fees from Galderma, Menlo Therapeutics, Novartis, and Trevi Therapeutics. SS is an investigator for Dermasence, Galderma, Kiniksa, Menlo Therapeutics, Trevi Therapeutics, Novartis, Sanofi, and Vanda Therapeutics; a consultant and/or member of the advisory board for Almirall, Beiersdorf, Bellus Health, Bionorica, Cara Therapeutics, Celgene, Clexio, DS Biopharma, Galderma, Kiniksa Menlo Therapeutics, Novartis, Sanofi, and Trevi Therapeutics.
